# Cancer Survivors' Experiences With and Preferences for Medical Information Disclosure and Advance Care Planning: An Online Survey Among Indonesian Cancer Support Groups

**DOI:** 10.1200/GO.23.00003

**Published:** 2023-04-12

**Authors:** Diah Martina, Rebecca Noerjani Angka, Rudi Putranto, Hamzah Shatri, Aru Wisaksono Sudoyo, Agnes van der Heide, Carin C.D. van der Rijt, Judith A.C. Rietjens

**Affiliations:** ^1^ Department of Medical Oncology, Erasmus MC Cancer Institute, University Medical Centre Rotterdam, the Netherlands; ^2^ Department of Public Health, Erasmus MC, University Medical Centre Rotterdam, the Netherlands; ^3^ Division of Psychosomatic and Palliative Medicine, Department of Internal Medicine, Faculty of Medicine Universitas Indonesia, Jakarta, Indonesia; ^4^ Cipto Mangunkusumo National General Hospital, Jakarta, Indonesia; ^5^ Indonesian Cancer Foundation Jakarta Chapter, Jakarta, Indonesia; ^6^ Division of Hematology and Medical Oncology, Department of Internal Medicine, Faculty of Medicine Universitas Indonesia, Indonesia; ^7^ Indonesian Cancer Foundation, Jakarta, Indonesia

## Abstract

To understand the experiences and preferences of Indonesian cancer survivors regarding medical information disclosure and advance care planning.

**METHODS:**

On the basis of systematic reviews of the scientific literature, qualitative studies, and expert-panel input, we developed an online survey that was distributed to nine cancer survivor support groups in Indonesia.

**RESULTS:**

A total of 1,030 valid responses were received. Most participants were younger than 60 years (92%), female (91%), married (78%), Muslim (75%), diagnosed with breast cancer (68%), highly educated (64%), and more than one year beyond diagnosis of their cancer. If diagnosed with a life-limiting illness, participants wished to be informed about their diagnosis (74%), disease severity (61%), estimated curability (81%), expected disease trajectory (66%), and estimated life expectancy (37%). Between 46%-69% of the participants wished to discuss four topics of advance care planning (end-of-life treatments, resuscitation, health care proxies, and what matters at the end of life); 21%-42% had done so. Of those who wished to discuss these topics, 36%-79% preferred to do so with their family members. The most important reasons for not being willing to engage in advance care planning were the desire to surrender to God's will and to focus on here and now.

**CONCLUSION:**

In a group of cancer survivors, most of them were highly educated, young, female, and diagnosed with breast cancer. Their preferences for medical information and advance care planning varied, with the majority wishing for information and involvement in advance care planning. Culturally sensitive advance care planning involves health care professionals eliciting individuals' preferences for medical information disclosure and discussing different topics in advance care planning conversations.

## BACKGROUND

Advance care planning enables individuals to define, discuss, and record their goals and preferences for future medical treatment and care. Its aim is to ensure that their treatment and care are aligned with these goals and preferences and in situations in which patients later lose their mental capacity.^1^ To allow meaningful engagement in advance care planning, individuals need sufficient knowledge of their medical condition.^2^ However, both advance care planning and disclosure of medical information are culturally sensitive.^2,3^ For instance, the common partial or nondisclosure of bad news surrounding life-limiting illnesses in Asia may limit patients' understanding of their illness.^2,3^ Similarly, their uptake of advance care planning can be limited by beliefs about death and dying or by the role of family in decision making.^2,3^

**CONTEXT**

**Key Objective**
To understand cancer survivors' perspectives on medical information disclosure and advance care planning in Indonesia, an Asian lower middle-income country with religiously devout populations and a collectivist culture.
**Knowledge Generated**
If diagnosed with a life-limiting illness, the majority of participants wished to be informed about their illness—preferably by their health care professionals—and were willing to engage in advance care planning, particularly with their family members and before they became terminally ill. However, relatively few of them wished to know about their estimated life expectancy, discussed resuscitation, documented the conversations, or had engaged in advance care planning conversations.
**Relevance**
Culturally sensitive advance care planning requires health care professionals to elicit and tailor their approach on the basis of individuals' preferences for medical information (types of information and way of communicating it) and preferences for discussing different topics in advance care planning conversations.

A declaration issued by a panel of Asian experts in 2019 recommended that studies on advance care planning prioritize cultural sensitivity.^4^ To our knowledge, to date, however, most Asian studies have been performed in high-income countries^2,3,5^ and have not taken into account the combination of collectiveness (a culture that prioritizes the group over the individual) and religiosity (self-identified religious importance) that are central to medical decision making in low- and middle-income countries such as Indonesia.^6^ Evidence suggests that people living in low- and middle-income countries tend to be more collectivistic and place higher importance on religion in their lives than those living in high-income countries.^7,8^ In Indonesia, advance care planning is not widely recognized as a useful concept, and do-not-resuscitate (DNR) forms are the only recognized type of advance care planning document.^5^

Although it is difficult for many people in Indonesia to talk about death and dying,^6^ cancer survivors have been confronted with potential life-limiting illnesses and their possible recurrence. Therefore, they may have contemplated an adverse future and/or engaged in advance care planning. This study aimed to elicit some of these survivors' experiences and perspectives on the provision of medical information and advance care planning. We particularly focused on members of cancer support groups who were open to participation in this study.^9,10^

## METHODS

### Study Design and Setting

An open web-based survey of Indonesian cancer survivors was conducted between July and September 2021. The results were reported according to the Checklist for Reporting Results of Internet E-Surveys (CHERRIES).^11^

### Population

**FIG 1:**
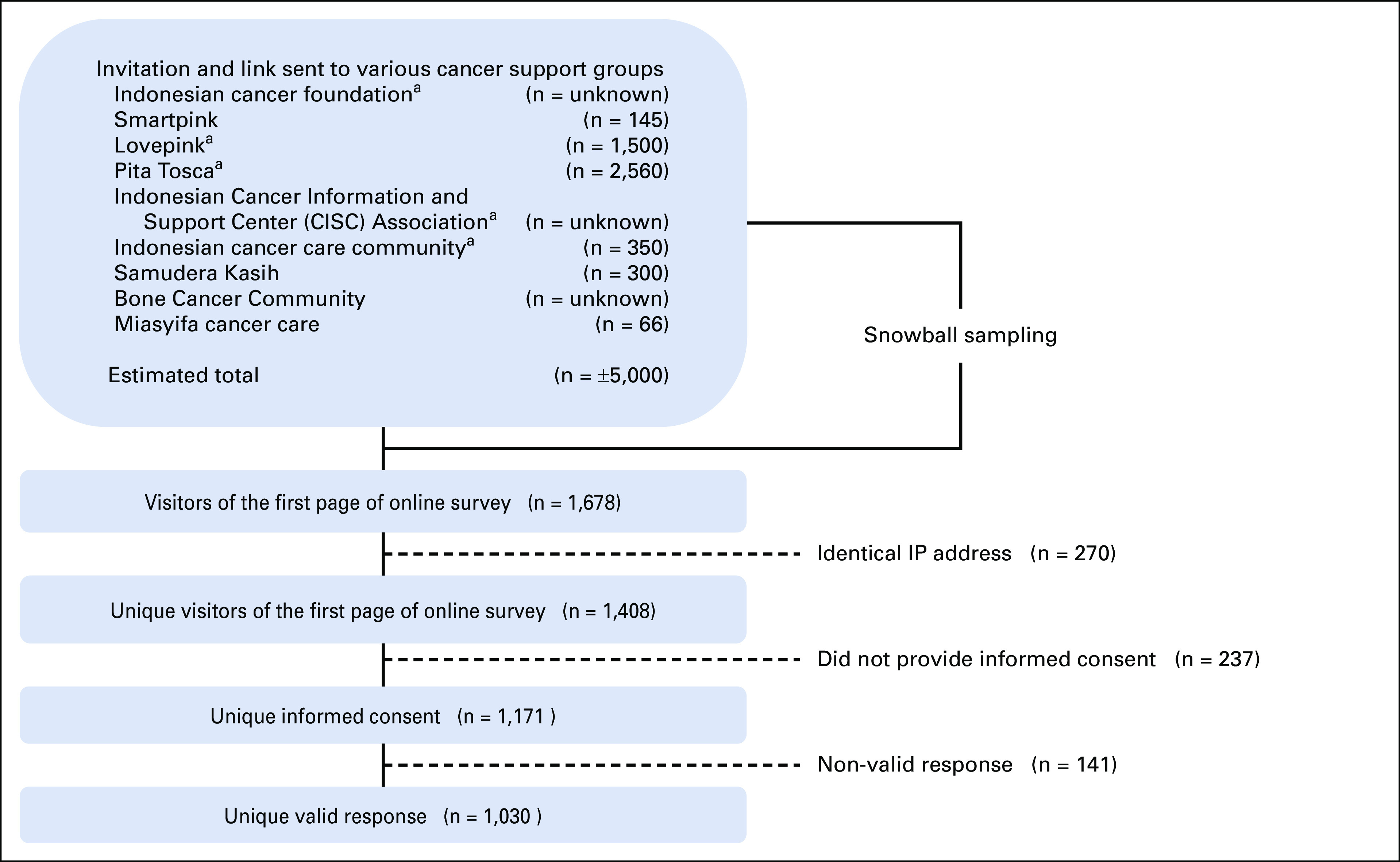
Flowchart of inclusion. ^a^Cancer support group with national member coverage. CISC, cancer information and support center.

We conceptualized a cancer survivor as any individual who has been cured, is in remission, or has active cancer.^12^ We included individuals who (1) were age 18 years or older, (2) had been diagnosed with a solid or hematologic malignancy at least 6 months before completing the survey, and (3) agreed to participate in the survey and provided informed consent for the study. Considering that Indonesia has no national registry of cancer survivors, we decided to conduct convenience sampling by approaching nine cancer survivor support groups in Indonesia, including five groups with national coverage (Fig 1). Two of the nine groups were breast cancer survivors. All the cancer survivor support groups agreed to distribute the survey to their members.

### Data Collection

This study was conducted during the COVID-19 pandemic in Indonesia in 2021. Because of the implementation of semilockdown measures,^13^ we used an online platform for our survey.

The survey was advertised by distributing the study announcement and a link to the online survey (Data Supplement) through WhatsApp groups of cancer survivor support groups. WhatsApp is the most popular instant messaging platform and the most frequently used group-based communication tool in Indonesia.^14,15^ We also used the snowball sampling method; we asked participants to send the WhatsApp link to other potential participants for the study. This method was used to sample participants in difficult-to-reach or hidden populations.^16^

### Questionnaire Development and Pretesting

First, D.M., J.A.C.R., C.C.D.v.d.R., and A.v.d.H. developed a questionnaire (Data Supplement) on the basis of previous systematic reviews on ACP in Asia,^2,3^ qualitative studies in Indonesia,^6,17^ and a cross-sectional survey of the Dutch general population.^18^ The questionnaire was translated from English into Bahasa, the main Indonesian language. To ensure that it would maintain the meaning of the original version, a bilingual researcher first forward translated every item of the questionnaire into Bahasa. It was then back translated by an independent bilingual researcher who was blinded to the original questionnaire. A comparison of the original and back-translated questionnaires by D.M. revealed no differences in meaning. Next, the translated questionnaire was sent to 10 Indonesian experts: two medical oncologists, three palliative care physicians, two oncology nurses, two palliative care nurses, and one psychologist for review. On the basis of their feedback, revisions were made to clarify the meaning and make the text easier to understand. Finally, the questionnaire was piloted among 15 cancer survivors to determine whether they found the questions clear and whether there were technical difficulties in completing the electronic questionnaire. On the basis of their feedback, final adjustments were made.

The questions were intended to assess the following: (1) participants' sociodemographic characteristics (age, sex, marital status, living situation, education, employment status, monthly household income, medical insurance, and religion); (2) their clinical characteristics (self-perceived health status, cancer diagnosis, time since initial cancer diagnosis, treatment for cancer, time of the last cancer treatment, and comorbid illnesses); (3) their experiences with and perspectives on the provision of medical information related to cancer and life-limiting illnesses; and (4) their experiences with and perspectives on advance care planning.

### Data Management and Analysis

The data were collected using a secured online survey system LimeSurvey^19^ and locked before the data analysis. Given the open recruitment of the participants, the response rate could not be determined. On the basis of the CHERRIES framework, we calculated the participation rate by dividing the total number of unique participants who provided informed consent by the total number of unique visitors who visited the survey landing page.^11^ To minimize duplicate responses, we performed an IP check. In the case of duplicate IP addresses, we included only the first completed survey for further analysis. To protect against unauthorized access, pseudoanonymized information was collected and stored where it was accessible only to the primary investigator. A response was considered valid and analyzed when it reached the question about advance care planning (question 19 of 25). We further determined the completion rate by dividing the number of participants who responded to all the questions by the total number of unique participants who had completed the informed consent.^11^

We used descriptive analyses to describe cancer survivors' demographic and clinical characteristics and their perspectives on information disclosure and advance care planning. Depending on the data distribution, data in numerical values are displayed as means and standard deviations or medians and ranges. SPSS v.25 (IBM Corp, Armonk, NY) was used for data analysis.

### Ethics Approval

This study was approved by the Ethical Committee of the Faculty of Medicine at Universitas Indonesia—Cipto Mangunkusumo Hospital on May 10th, 2021 (KET-453/UN2.F1/ETIK/PPM.00.02/2021).

### Consent to Participate

Participants were informed of the purpose and design of the study and provided informed consent on the first page of the online survey (Data Supplement). Participants who completed the survey were offered a token of appreciation in the form of a data bundle worth around 3.5 USD.

### Consent for Publication

The authors affirm that participants provided informed consent for publication.

## RESULTS

### Characteristics of the Participants

The first page of the online survey was visited 1,678 times (Fig 1). After removing identical IP addresses (n = 270) and questionnaires where the informed consent question was not answered (n = 237), we obtained 1,171 unique responses (participation rate = 83%). Subsequently, we removed invalid responses or responses that did not answer the first question related to advance care planning (question 19, n = 141). This left 1,030 valid responses; 960 participants completed all questions in the survey (completion rate = 82%). Our analysis was based on 1,030 valid responses.

Most participants (Table 1) were younger than 60 years (92%), female (91%), married (78%), and Muslim (75%), considered themselves to be in a healthy state (84%), had been diagnosed with breast cancer (68%), and had completed higher education (64%). Seventy seven percent of the participants were more than one year beyond their diagnosis of cancer, 19% were more than 5 years beyond diagnosis, 47% had completed cancer treatment, and 41% were still receiving such treatment when they participated in the survey.

**TABLE 1:**
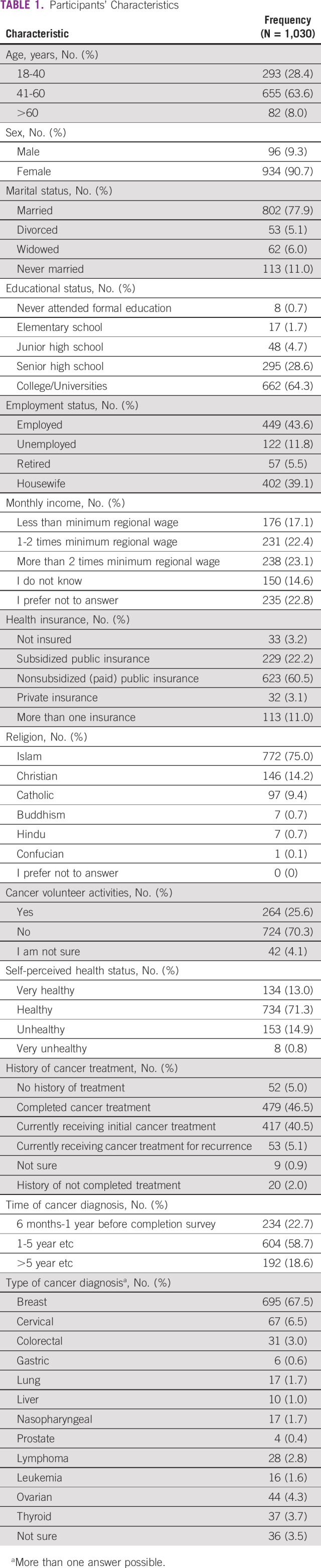
Participants' Characteristics

### Participants’ Experiences With and Preferences Regarding the Provision of Information Related to Serious Illness

Ninety-four percent of the participants were informed about their initial cancer diagnosis by a physician and 3% by family members (Table 2). Although most participants were informed about their type of cancer (90%) and stage (68%), few had been informed about the curability of their cancer (54%), the risk of recurrence (37%), or their life expectancy (19%). When asked what information they would wish to receive if they were diagnosed with a life-limiting illness, 81% of participants answered that they would appreciate information on the curability of the disease, 66% information about the expected disease trajectory, and 37% information about their life expectancy. Seventy-five percent of the participants who were willing to be informed about their illness wished to be informed directly by their physician with or without the presence of their family members.

**TABLE 2:**
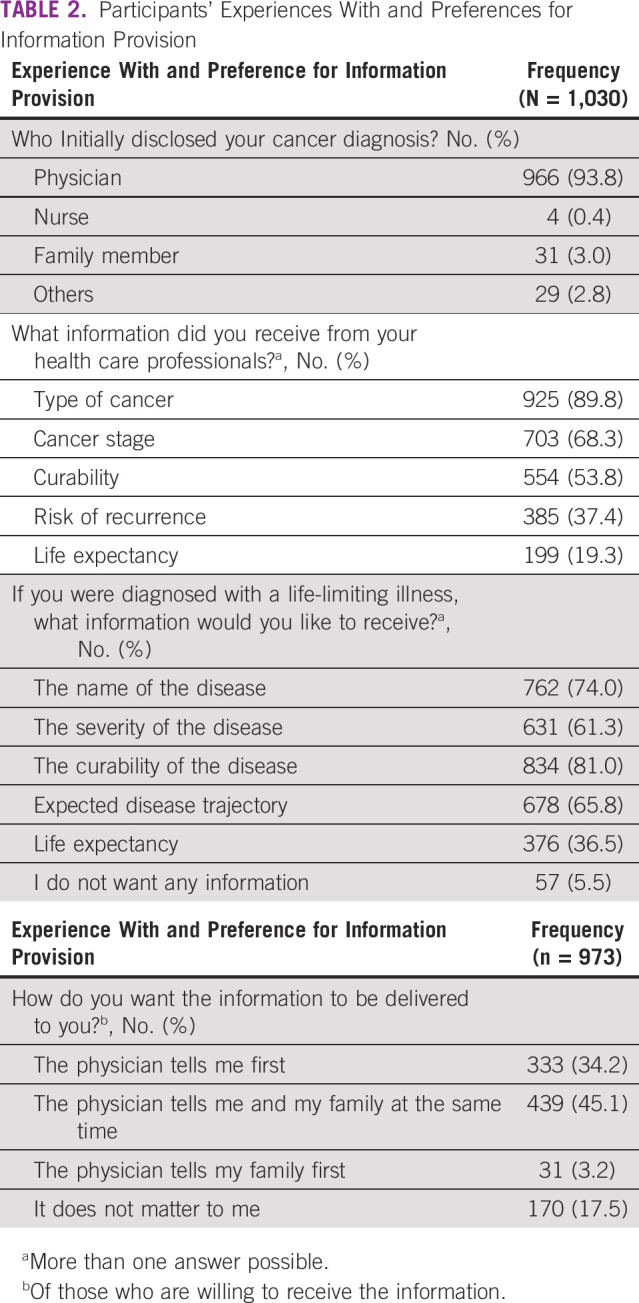
Participants' Experiences With and Preferences for Information Provision

### Participants’ Experiences With and Preferences Regarding Advance Care Planning

**FIG 2:**
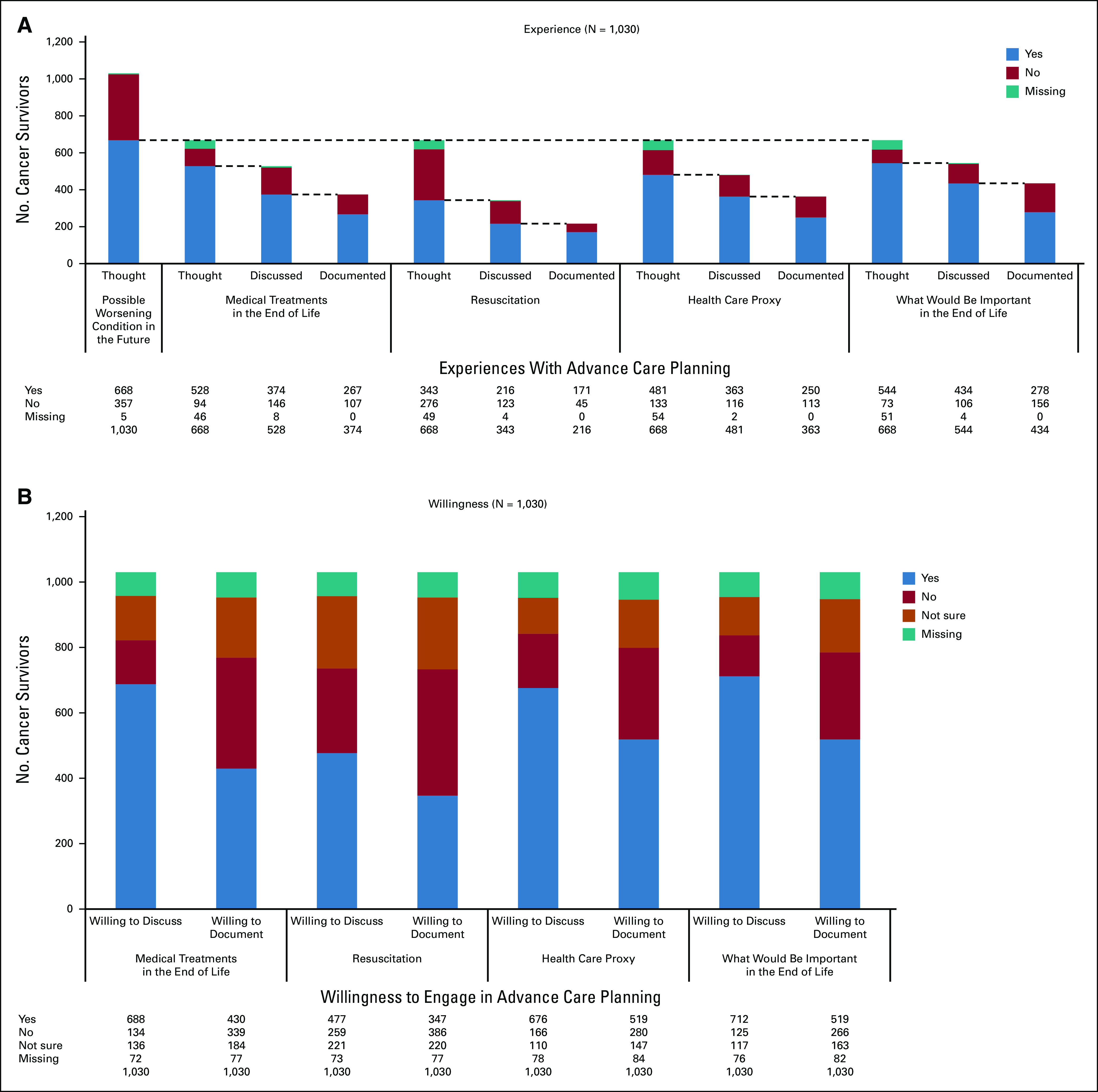
Cancer survivors' experiences with and willingness to engage in advance care planning. (A) Cancer survivors' experiences with advance care planning. The dashed lines show that those who answer “Yes” to the first questions will be asked the next ones. (B) Cancer survivors' willingness to engage in advance care planning.

Sixty-five percent of the 1,030 participants (Fig 2A) had thought about the possible future worsening of their condition. More specifically, 51% had thought about the medical treatments they would prefer at the end of life, 33% about resuscitation, 47% about health care proxies, and 53% about what would be important for them at the end of life. Fewer of them had discussed these topics with others (36%, 21%, 35%, and 42%, respectively) or had documented their preferences in an advance directive (26%, 12%, 24%, and 27%, respectively).

Approximately two-thirds of the 1,030 participants (Fig 2B) indicated that they were willing to discuss their medical treatment at the end of life (67%), health care proxies (66%), and what would be important for them at the end of life (69%). Fewer participants (46%) were willing to discuss resuscitation. Half of the participants were willing to make written statements about who would be their health care proxy and what would be important for them at the end of life. Fewer were willing to document preferences for medical treatment and care at the end of life (42%) and whether or when they would opt for resuscitation (34%).

Over half of those who had thought about advance care planning topics but had never discussed them with others were willing to do so (55%-69%; Data Supplement). Less than half of those who had not documented their preferences for future treatment and care (34%-50%) were willing to do so (Data Supplement).

Regarding the initiation of advance care planning conversations, 31% of all participants (Data Supplement) wished to do so when they were still healthy, 30% when diagnosed with an incurable illness, and 5% when their life expectancy was <6 months. Seventeen percent had no clear idea of their preferences, and 9% wanted not to have an advance care planning conversation.

Most of the participants who had discussed elements of advance care planning (Data Supplement) had discussed them with their family members (78%-96%), some of them with (13%-38%) and without (40%-84%) healthcare professionals. Similarly, most participants (50%-97%) were willing to discuss these topics with their family members, some of them with (14%-50%) and without (36%-79%) health care professionals.

As shown in the Data Supplement, the reasons for willingness to engage in advance care planning were that the family would then understand the participants' values, wishes, and preferences for end-of-life care (61%); that participants wanted to decide on their own future care (60%); that they wanted to avoid end-of-life suffering (46%); and that they did not want to put the burden of decision making on their family (46%). Frequently cited reasons for not wanting to engage in advance care planning were the belief that it is more important to surrender to God's will than to have control over the future (53%), belief that death is a natural event (40%), and desire to focus on the present (40%).

## DISCUSSION

More than half of the participating cancer survivors in this study were younger than 60 years, female, had completed higher education, were diagnosed with breast cancer, and were more than 1 year from diagnosis of their cancer. They wished to be informed about their illnesses and were willing to engage in advance care planning. Although many participants were willing to discuss several elements of advance care planning, they had not yet discussed them all. The majority of those willing to engage in advance care planning preferred to do so before becoming terminally ill. The most important reasons for not wanting to engage in advance care planning were the desire to surrender to God's will, the belief that people should not intervene in the natural process of dying, and the wish to focus on here and now.

Our study showed that three quarters of the participants wished to be informed about their illness by their physicians rather than by family caregivers. Whereas family caregivers in a previous study considered mediated information provision to be a sensitive way of delivering bad news,^17^ the majority of cancer survivors in the current study preferred information provision not to be mediated. A minority wished to be informed about their estimated life expectancies. A previous study showed that some patients considered such information irrelevant, believing life to be God's sole authority.^17^ Others would avoid such information to preserve hope.^17^ These findings demonstrate that accurate prognostic awareness can have both benefits and disadvantages. On the one hand such awareness may promote informed and value-based decision making, thus enabling the attainment of goal-concordant care.^20,21^ On the other hand, prognostic awareness has been found to be associated with a worse quality of life, higher anxiety and depression levels, and a higher sense of burden.^22-24^ Patients with cancer worldwide have various preferences for prognostic disclosure, with people preferring a broad indication of prognosis rather than exact quantitative information.^25^ Accordingly, before medical information is disclosed to a patient, it is important to determine the information they prefer.

Nearly 70% of the participants in this study who had thought about one or more aspects of advance care planning but had never discussed them with others were actually willing to do so. Furthermore, the majority of participants who had engaged or would be willing to engage in advance care planning conversations had done so or would do so with their family members, sometimes without the presence of health care professionals. Therefore, most cancer survivors seem to consider family involvement in advance care planning essential. Prior evidence suggests that patients from countries with collectivist cultures more often wish to involve their family members in such conversations than patients from Western countries.^26^ Therefore, supporting family members' engagement in advance care planning could indirectly facilitate patient engagement.^27^ We also found that one third of our participants wished to initiate advance care planning conversations on being diagnosed with an incurable illness and one-third even earlier, when they were still healthy. These findings indicate the scope for extending advance care planning initiatives from health care settings to community settings (eg, within families, faith groups, or cancer support groups). Cancer support groups might serve as an effective platform for promoting advance care planning among their members, considering their effectiveness in fostering confidence and self-efficacy in decision making through the provision of a safe, trusting, and empowering environment.^28^

Although a DNR order is currently the only available form of an advance directive in Indonesia,^5^ our findings show that few cancer survivors were willing to discuss resuscitation. Resuscitation may be a relatively difficult topic in advance care planning conversations,^29^ especially in Asia, where death and dying are taboo topics.^3^ Indonesian health care professionals should, therefore, be educated to approach advance care planning as a discussion that not only addresses resuscitation but also value exploration. Individuals' reluctance to consider certain topics does not necessarily exclude them from engaging in advance care planning. Instead, engaging them in a topic they are ready to discuss is necessary to creating meaningful conversations and a trusting relationship between patient and health care professionals, which may further facilitate their readiness to talk about other difficult topics.^29^ Our finding of this reluctance also supports the need for a wider conceptualization of advance care planning as a process of value exploration, rather than merely conversations about future treatment planning.

To our knowledge, this is the first survey to explore Asian cancer survivors' perspectives on advance care planning. Second, our study included a large number of participants: a wide variety of major Indonesian cancer support groups, five of which covered national membership and high participation and completion rates. Third, our methodology allowed us to evaluate sensitive topics in a selective population, which was presumably more open and motivated to engage in discussing culturally sensitive topics regardless of the COVID-19 pandemic situation.

Two main limitations need to be considered when interpreting this study. First, our study is an open web-based survey involving convenience sampling, which can be subject to considerable bias because of the self-selection of participants who needed to be able to access the Internet. Second, on the basis of patients' self-perceived health status and the low percentage (5.1%) of patients who were receiving cancer treatment for recurrence, many patients may have been treated with curative intent, limiting its generalizability to patients in their last 12 months of life. Further research is needed to examine preferences for medical information and advance care planning across various cultures while taking into account the patients' disease trajectory and prognosis. The high representation of young, female, educated breast cancer survivors in our study is consistent with the characteristics of online survey participants in prior studies of cancer support groups.^9^ Therefore, the findings of this study may not be generalizable to cancer survivors with other characteristics.

Second, this study may be subject to recall bias and inaccurate interpretation of medical information. However, our survey focused on exploring participants' perspectives and preferences rather than their accurate understanding of their own medical conditions.

The majority of the participating members of the Indonesian cancer survivor support groups were highly educated, motivated, female, relatively young, and more than one year beyond diagnosis of their cancer. The participants' preferences for medical information and involvement in advance care planning varied widely. Those who were willing to engage in advance care planning rarely had done so. Culturally sensitive advance care planning involves health care professionals eliciting individuals' preferences for medical information disclosure and engagement in discussing different topics in advance care planning conversations.

## References

[1] Rietjens JAC Sudore RL Connolly M et al Definition and recommendations for advance care planning: An international consensus supported by the European Association for Palliative Care Lancet Oncol 18 e543 e551 2017 28884703 10.1016/S1470-2045(17)30582-X

[2] Martina D Geerse OP Lin CP et al Asian patients’ perspectives on advance care planning: A mixed-method systematic review and conceptual framework Palliat Med 35 1776 1792 2021 34488509 10.1177/02692163211042530 PMC8637390

[3] Martina D Lin CP Kristanti MS et al Advance care planning in Asia: A systematic narrative review of healthcare professionals’ knowledge, attitude, and experience J Am Med Directors Assoc 22 349.e1 349.e28 2021 10.1016/j.jamda.2020.12.018 33421371

[4] Lin CP Cheng SY Mori M et al 2019 Taipei declaration on advance care planning: A cultural adaptation of end-of-life care discussion J Palliat Med 22 1175 1177 2019 31268393 10.1089/jpm.2019.0247

[5] Cheng SY Lin CP Chan HYL et al Advance care planning in Asian culture Jpn J Clin Oncol 50 976 989 2020 32761078 10.1093/jjco/hyaa131

[6] Martina D Kustanti CY Dewantari R et al Opportunities and challenges for advance care planning in strongly religious family-centric societies: A focus group study of Indonesian cancer-care professionals BMC Palliat Care 21 110 2022 35729537 10.1186/s12904-022-01002-6 PMC9215088

[7] Pew Research Center The Global God Divide 2020 https://www.pewresearch.org/global/2020/07/20/the-global-god-divide/

[8] Haslam D Mejia A Thomson D et al Self-regulation in low- and middle-income countries: Challenges and future directions Clin Child Fam Psychol Rev 22 104 117 2019 30725308 10.1007/s10567-019-00278-0

[9] van Eenbergen MC van de Poll-Franse LV Heine P et al The impact of participation in online cancer communities on patient reported outcomes: Systematic review JMIR cancer 3 e15 2017 28958985 10.2196/cancer.7312 PMC5639205

[10] Grande GE Myers LB Sutton SR How do patients who participate in cancer support groups differ from those who do not? Psycho-Oncology 15 321 334 2006 16106472 10.1002/pon.956

[11] Eysenbach G Improving the quality of web surveys: The checklist for reporting results of Internet E-Surveys (CHERRIES) J Med Internet Res 6 e34 2004 15471760 10.2196/jmir.6.3.e34 PMC1550605

[12] Mayer DK Nasso SF Earp JA Defining cancer survivors, their needs, and perspectives on survivorship health care in the USA Lancet Oncol 18 e11 e18 2017 28049573 10.1016/S1470-2045(16)30573-3

[13] UN Office for the Coordination of Humanitarian Affairs Situation Update: Response to COVID-19 in Indonesia (As of 8 September 2021) 2021 https://reliefweb.int/report/indonesia/situation-update-response-covid-19-indonesia-8-september-2021

[14] Mulyono H Suryoputro G Jamil SR The application of WhatsApp to support online learning during the COVID-19 pandemic in Indonesia Heliyon 7 e07853 2021 34485735 10.1016/j.heliyon.2021.e07853 PMC8405994

[15] Team G Indonesia's Social Media Landscape: An Overview 2019 https://greenhouse.co/blog/indonesias-social-media-landscape-an-overview/

[16] Brewerton PMML Sampling considerations Organizational Research Methods London, United Kingdom SAGE Publications, Ltd 2001 114 121

[17] Martina D Kustanti CY Dewantari R et al Advance care planning for patients with cancer and family caregivers in Indonesia: A qualitative study BMC Palliat Care 21 204 2022 36414948 10.1186/s12904-022-01086-0 PMC9682799

[18] Raijmakers NJH Rietjens JA Kouwenhoven PS et al Involvement of the Dutch general population in advance care planning: A cross-sectional survey J Palliat Med 16 1055 1061 2013 23767949 10.1089/jpm.2012.0555

[19] Limesurvey GmbH LimeSurvey: An Open Source Survey Tool Hamburg, Germany LimeSurvey GmbH 2003

[20] Wen FH Chen JS Chang WC et al Accurate prognostic awareness and preference states influence the concordance between terminally ill cancer patients' states of preferred and received life-sustaining treatments in the last 6 months of life Palliat Med 2019 33 1069 1079 31185815 10.1177/0269216319853488

[21] Tang ST Chen CH Wen FH et al. Accurate prognostic awareness facilitates, whereas better quality of life and more anxiety Symptoms hinder end-of-life care discussions: A longitudinal survey study in terminally ill cancer patients' last six months of life J Pain Symptom Manage 55 1068 1076 2018 29289656 10.1016/j.jpainsymman.2017.12.485

[22] Tang ST Chang WC Chen JS et al. Associations of prognostic awareness/acceptance with psychological distress, existential suffering, and quality of life in terminally ill cancer patients' last year of life Psycho-Oncology 2016 25 455 462 26283000 10.1002/pon.3943

[23] Nipp RD Greer JA El-Jawahri A et al. Coping and prognostic awareness in patients with advanced cancer J Clin Oncol 35 2551 2557 2017 28574777 10.1200/JCO.2016.71.3404 PMC5536163

[24] Vlckova K Tuckova A Polakova K et al. Factors associated with prognostic awareness in patients with cancer: A systematic review Psycho-Oncology 29 990 1003 2020 32285580 10.1002/pon.5385

[25] Innes S Payne S Advanced cancer patients' prognostic information preferences: A review Palliat Med 23 29 39 2009 18952746 10.1177/0269216308098799

[26] Fujimori M Uchitomi Y Preferences of cancer patients regarding communication of bad news: A systematic literature review Jpn J Clin Oncol 39 201 216 2009 19190099 10.1093/jjco/hyn159

[27] Kishino M Ellis-Smith C Afolabi O et al Family involvement in advance care planning for people living with advanced cancer: A systematic mixed-methods review Palliat Med 36 462 477 2022 34989274 10.1177/02692163211068282 PMC8972955

[28] Gupta T Schapira L Online communities as Sources of peer support for people living with cancer: A commentary JCO Oncol Pract 14 725 730 2018 10.1200/JOP.18.00261 30335558

[29] Zwakman M Milota MM van der Heide A et al Unraveling patients’ readiness in advance care planning conversations: A qualitative study as part of the ACTION study Support Care Cancer 29 2917 2929 2021 33001268 10.1007/s00520-020-05799-x PMC8062377

